# Role of the amygdala opioid system in the effects of stress on the post-learning sleep patterns of male Wistar rats

**DOI:** 10.22038/ijbms.2024.79291.17266

**Published:** 2025

**Authors:** Mehdi Graily-Afra, Farideh Bahrami, Zahra Bahari, Hedayat Sahraei, Zeinab Shankayi, Ali Gharib

**Affiliations:** 1 Neuroscience Research Center, Baqiyatallah University of Medical Sciences, Tehran, Tehran, Iran; 2 Department of Physiology and Medical Physics, School of Medicine, Baqiyatallah University of Medical Sciences, Tehran, Iran; 3 Students’ Research Committee, Baqiyatallah University of Medical Sciences, Tehran, Iran

**Keywords:** Brain-derived neurotrophic - factor (BDNF), Baso lateral amygdala (BLA), Learning, Memory, Naloxone, Sleep, Stress

## Abstract

**Objective(s):**

Three physiological processes interact: sleep, learning, and stress. It is essential to understand how stress affects and interacts with the link between sleep, learning, and memory since it has long been recognized that sleep plays a crucial role in memory consolidation and learning. Through naloxone injection in the baso lateral amygdala (BLA), this study intends to shed light on the interactions between stress, learning, and sleep, as well as the function of the opioid system and its impact on brain-derived neurotrophic factor (BDNF) production in the hippocampus.

**Materials and Methods:**

Male Wistar rats (n=77) in eleven groups were implanted with electroencephalogram (EEG) and electromyography (EMG) recording electrodes, and the BLA area was bilaterally cannulated. Recordings of Rapid Eye Movement (REM) and Non-Rapid Eye Movement (NREM) sleep and wakefulness steps were made for the three hours prior to and three hours following the implementation of the immobility stress protocol and learning with the Barnes maze for three consecutive days. Also, the animals’ memory was tasted 48 hr later. Before the stress and learning procedure, naloxone was injected into each BLA three times in a row at a dosage of 0.05 μg or 0.1 μg in a volume of 0.5 μl. A molecular biomarker of learning and stress, BDNF, was also examined.

**Results:**

The study demonstrated that the immobility stress model lowers REM and NREM sleep. On the other hand, putting the learning technique into practice results in more REM and NREM sleep, and stress situations do not stop this rise after learning. Naloxone injections in the BLA region also enhance learning and memory, preventing stress-related REM and NREM sleep loss. Additionally, stress lowers BDNF expression in the hippocampal region. BDNF expression rises in the hippocampus throughout the learning process, and naloxone administration in the BLA area also raises BDNF expression in the hippocampus.

**Conclusion:**

Stress generally reduces REM, NREM, and BDNF expression in the hippocampal region. Under stress, using the learning protocol increases REM, NREM sleep, and BDNF. Naloxone injection in BLA improves memory and learning, reducing stress-induced memory loss.

## Introduction

The body’s internal clock regulates sleep as a biological circadian rhythm. Numerous studies imply that memory and learning are influenced by sleep on a variety of levels. Some research demonstrates that REM sleep enhances memory, while others reveal that the beginning part of the night with slow-wave sleep  (SWS) significantly impacts memory consolidation (1).   Memory and learning are closely related to stress, and chronic and acute stress can also affect sleep structure and circadian rhythms (2). Stress decreases SWS sleep and prevents REM sleep, according to studies. After the stress is reduced, there are also notable alterations in SWS and REM sleep. Changes in sleep are influenced by the type and length of stressors and, perhaps most crucially, by the person’s capacity to handle stress. Opioid peptides also affect learning and memory (3). Studies have shown that acute and chronic injections of morphine before and after exercise impair learning. Consequently, administering naloxone and naltrexone can enhance memory stabilization (4).

The baso lateral amygdala (BLA) nucleus is one of the most prominent amygdala nuclei involved in memory and learning due to its close connections with the hippocampus (5). On the other hand, this nucleus has significant involvement in the stress process and affects the duration of sleep and wakefulness (6). Long-term memory involves a cascade of protein synthesis and neurotrophic factors, especially the brain-derived neurotrophic factor (BDNF) (7). In REM sleep, protein expression and electrical activity of pyramidal cells in the amygdala increase (8). Studies show that BDNF in the hippocampus is required during and an hour after training to build long-term memory. Another research study found that administering morphine after exercise reduced memory but administering BDNF to the CA1 area of the hippocampus corrected this effect (9). Chronic stress reduces the process of memory and learning and also reduces BDNF (8). Given that opioid receptors are involved in learning and memory and are also expressed in the BLA region, it is reasonable to assume that opioid receptors, due to stress, negatively affect memory and learning (10, 11). 

This research aims to explore the effects of sleep on brain-derived neurotrophic factor (BDNF) production in the hippocampus and the biochemical processes related to learning.

To mitigate the negative impacts of stress on sleep, we investigate how it affects sleep. The opioid receptor antagonist naloxone is administered into the basolateral amygdala (BLA) in order to achieve this. Therefore, we assess how stress, sleep, and learning interact through the opioid system. Sleep disruptions caused by stress should be minimized in order to promote better cognitive performance and memory consolidation. 

## Materials and Methods

**Figure 1:**
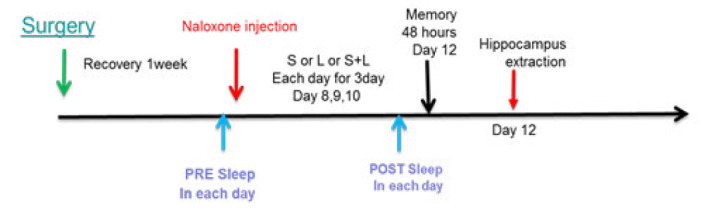
A scheme of experimental diagram in rat is presented for the time intervals

This study used 77 male Wistar rats aged between 2 to 3 months, weighing 180 and 250 g. The rats were divided into three groups: stress, learning, and learning with stress. Naloxone was injected in the BLA region (0.05 μg and 0.1 μg) (12) of 77 animals, divided into 11 groups. Three groups received a dose of 0.05 naloxone in each group (n=7) before stress, before learning, and before learning plus stress. Three groups received a dose of 0.1 μg naloxone (before stress, before learning, and before stress plus learning). The sham group was divided into three groups: stress (n=7), learning (n=7), and stress plus learning (n=7). They were injected with saline into the amygdala post-surgery. Two groups of 7 only received naloxone without stress and learning to evaluate the effect of naloxone. The animals were kept in cages with free access to food and water in a room at 20–25 °C temperature and a 12/12 light-dark cycle (Figure 1). 

### Electrophysiology and drug injection

EEG and EMG recordings were performed to diagnose the three REM and NREM sleep and wakefulness stages. After anesthetizing the animals with a mixture of ketamine (65 mg/kg) and xylazine (14 mg/kg), the animals were placed in the stereotaxic device. The location of the cannula in the BLA was calculated using the Paxinos Atlas, and the cannula was placed in both the left and right BLA. (A/P: -2.8 mm, M/L:5, D/V:8.5)(13). After inserting the guide cannula for naloxone injection for the EEG recording, three small stainless-steel screws were implanted in the skull area. Two screws served as positive and negative electrodes in the anterior parietal region, and the other screw served as the ground electrode in the posterior parietal region near the midline. A gap of about one centimeter was created in the animal’s occipital region to obtain the EMG signal. Two stainless steel wire electrodes were inserted into the neck muscles at a distance of one centimeter. Finally, all the electrodes were connected to the connector fixed on the surface of the animal’s skull using dental acrylic. EEG and EMG signals were amplified and filtered (EEG 0.5–40 Hz, EMG 1Hz- 1 kHz) with an e-wave system amplifier (Ruby Mind, NY, US) in the 1000 Hz sampling rate. 

To determine each animal’s baseline level of sleep, the sleep and wake signals were recorded for three days, every day from 8 to 11 in the morning. The REM and NREM sleep and wakefulness percentages were considered the animal’s baseline sleep by averaging these three days. Also, after implementing stress and learning protocols, sleep and wakefulness steps were recorded daily for at least three hours over three consecutive days. First, the record of the baseline sleep was recorded for three hours per day, then naloxone (0.05 and 0.1 μg) or saline as the vehicle was injected into the BLA (0.5 μl in each guide cannula). The injection was performed slowly over one minute, then one of the stress, learning, or both protocols was performed, and sleep was recorded for three hours per day for three consecutive days. The mean baseline and post-protocol sleep was then examined (14).

### Sleep wave analysis

**Figure 2:**
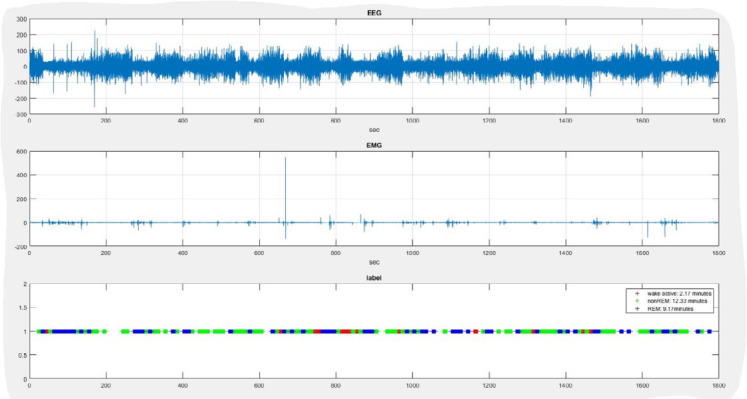
Sample of sleep and wakefulness analysis in a 30 min duration in MATLAB

EEG and EMG data spectral analysis was performed on all artifact-free epochs using the Fast Fourier Transform (FFT) algorithm (MATLAB, 2022a). According to previous research, the awake state is characterized by high EMG power (active wakefulness) and low delta power. NREM sleep showed low EMG power and high delta power, along with sleep spindles and K complexes. REM sleep had low delta and high theta power, as opposed to atonia or hypotonia in EMG. EEG and EMG spectral analysis determined the sleep stage from delta (1-4 Hz), theta (6-12 Hz), and total EMG power between 20–500 Hz. The power spectrum was computed with one-second sliding windows, moving by 0.5 sec each time, using FFT and multi-taper. The extracted features were clustered into three stages of sleep: wakefulness, NREM, and REM, using the C-means fuzzy clustering algorithm (FCM) with three clusters (Figure 2) (15-18).

### Multiple stress protocol

Multiple stress models, such as psychological and physical stress, have been used to induce stress in animals. The stress chamber, which had dimensions of 7x7x16 cm, was made of opaque Plexiglass to prevent the animal from seeing outside. The bottom of the box was covered with uneven stones, which created a narrow and uneven environment for the animal. The stress time was two hours on three consecutive days and was performed after recording the sleep baseline (19-21).

### Barnes maze protocol

Each animal was placed in the maze for 20 min on day zero. In this case, the target chamber was not located under any hole. After the adaptation phase, the learning process began by placing the animal in the maze’s center inside the primer cylinder (for 20 sec); after removing the primer cylinder, the animal was given 5 min to find the target chamber. If not, they were taken to the target chamber by hand and remained inside the chamber for 60 sec. The training steps included five trials per day for three consecutive days for memory analysis; 48 hours later, only one trial was performed. After each work with the Barnes maze, the whole surface was sterilized with a 70% alcohol solution. The time to reach the target chamber, the number of errors, and the distance traveled by the animal were examined to evaluate the results.

### BDNF measurement

The level of BDNF was measured using an ELISA test. At the end of the experiments, the animals were euthanized with a combination of Ketamine and Xylazine. The rats were decapitated 45 min following an intraperitoneal injection of 100 mg/kg Ketamine with 10 mg/kg Xylazine hydrochloride supplement, and the hippocampus was extracted. The dissected hippocampal tissue was placed in a microtube to which 80 μl of RIPA solution was added and kept at 4 °C for an hour. The samples were centrifuged for 15 min at 1500 rpm, and the supernatant was transferred to a new microtube. After adding 10 μl of BDNF antibody into each well, 50 μl of HRP was added, and samples were placed at 37 °C for one hour. The level of BDNF attached to each well was determined by incubating for 30 min with 50 µl each of chromogen A and chromogen B substrates. Following the addition of the STOP solution, the developed blue color was quantified using a spectrophotometer.

### Statistical analysis

SPSS version 22 was used to analyze the data. The baseline sleep limit was considered the average amount of REM sleep, NREM sleep, and wakefulness during these three days. To investigate the effect of stress and learning on REM, NREM sleep, and wakefulness, paired t-tests were performed to compare the variables within each animal before and after learning, stress, or both. First, to investigate the statistical differences between the study groups, the difference between REM, NREM sleep, and wakefulness before and after stress or learning was obtained. Then, a two-way analysis of variance test was employed to analyze the differences among these groups based on the implemented dose of naloxone. One-way analysis of variance was used to compare learning and memory on different days and memory test days between other groups. Values ​​were considered significant at *P*<0.05.

### Ethical consideration

This study has ethical approval from Baqiyatallah University of Medical Sciences (IR.BMSU.REC.1398.006) and complies with NIH Animal Care Protocols and Guidelines.

## Results

### The relationship between stress and sleep under naloxone treatment

**Figure 3:**
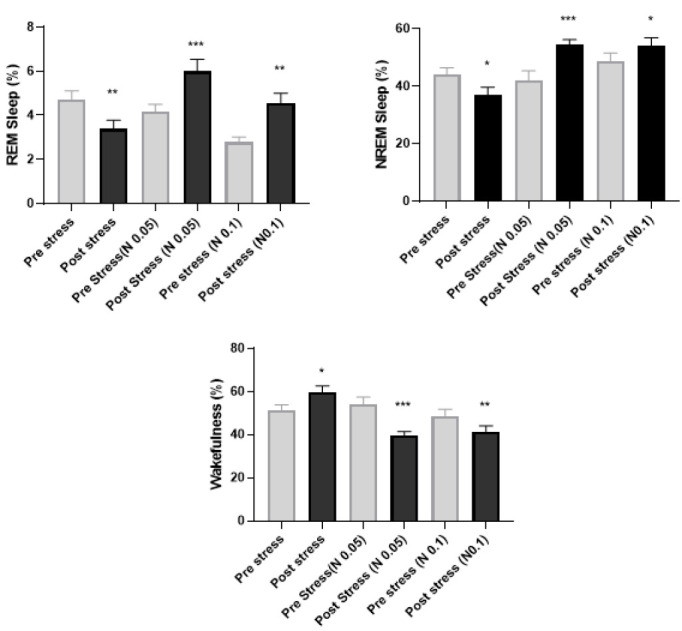
The graph shows the changes in REM and NREM sleep and wakefulness before and after stress in rat

The results showed a significant decrease in REM sleep when compared to the control group (*P*<0.01) as well as decreased NREM sleep (*P*<0.05) in stressed rats. Additionally, wakefulness was increased (*P*<0.05); in contrast, the results indicated that bilateral injection of naloxone (0.05 and 0.1 μg) in the basolateral amygdala significantly increased REM (*P*<0.001, *P*<0.01) and NREM (*P*<0.01,* P*<0.05) sleep values despite the presence of stress. It also significantly decreased wakefulness (*P*<0.01, *P*<0.05) (Figure 3).

### Relationship between learning and sleep under naloxone treatment

**Figure 4:**
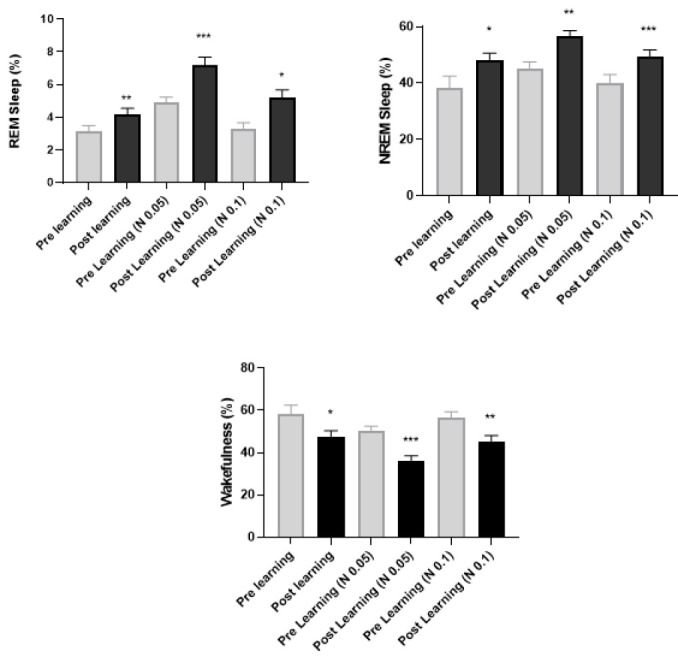
The graph shows the changes in REM and NREM sleep and wakefulness before and after learning in rat

The results showed that the implementation of the learning protocol leads to a significant increase in REM (*P*<0.01) and NREM sleep (*P*<0.05). In contrast, the total wakefulness of the animal after the learning process was significantly reduced (*P*<0.05) and also indicated that bilateral injection of naloxone (0.05 and 0.1 μg) in the basolateral amygdala accompanied by the learning protocol resulted in increased REM and NREM sleep (*P*<0.001 and *P*<0.05) and decreased awakening significantly (*P*<0.001 and *P*<0.01) (Figure 4).

### Sleep alteration after learning and stress procedures under naloxone treatment

**Figure 5:**
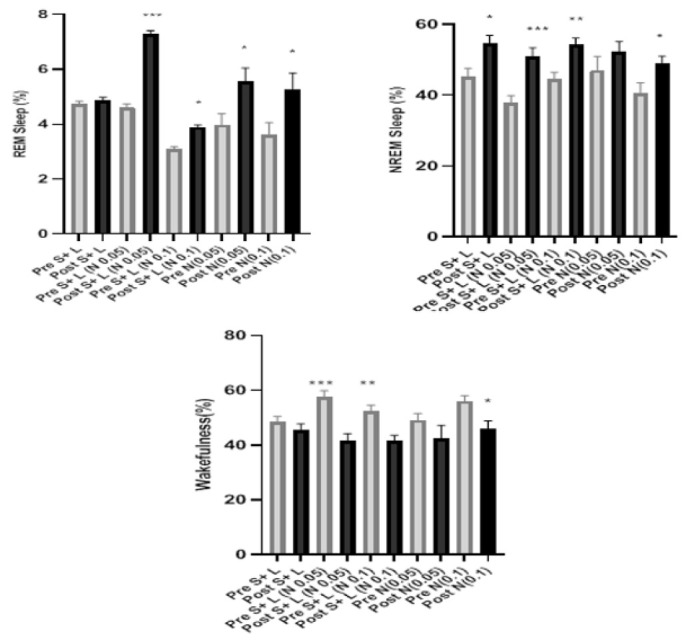
A comparison of REM, NREM sleep, and wakefulness before and after learning with learning and stress (L+S) and naloxone injections in two doses is shown in rat

The results showed that despite the stress implementing procedure, the learning process still led to a significant increase in NREM sleep (*P*<0.05) but had no significant effect on REM sleep. Overall, wakefulness was reduced, though not significantly. Moreover, bilateral injection of naloxone (0.05 and 0.1μg) in the basolateral amygdala, along with the entry of animals into stress and learning protocols, led to a significant increase in REM (*P*<0.001 and *P*<0.05) and NREM sleep (*P*<0.001 and *P*<0.01) and also a considerable decrease in wakefulness (*P*<0.01 for both doses) (Figure 5). The effects of naloxone 0.05 and 0.1 μg doses on sleep stages have been depicted in Figure 3. 

### An evaluation of naloxone dose dependency and changes in sleep and wakefulness among different groups

**Figure 6:**
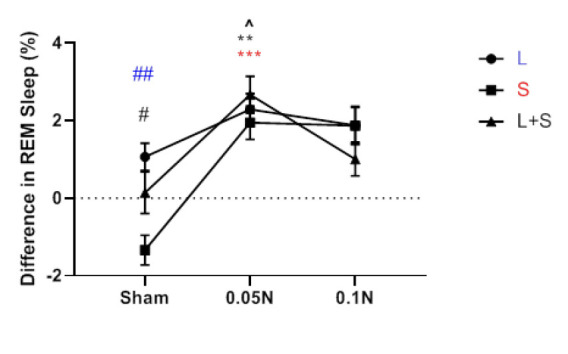
A comparison chart of the difference between REM sleep values before and after the implementation of learning protocols (L) and stress (S) and learning with stress (L + S), as well as the difference between REM sleep values with naloxone injection of 0.05 μg and 0.1 μg in these groups of rats with Each other (n=7)

**Figure 7:**
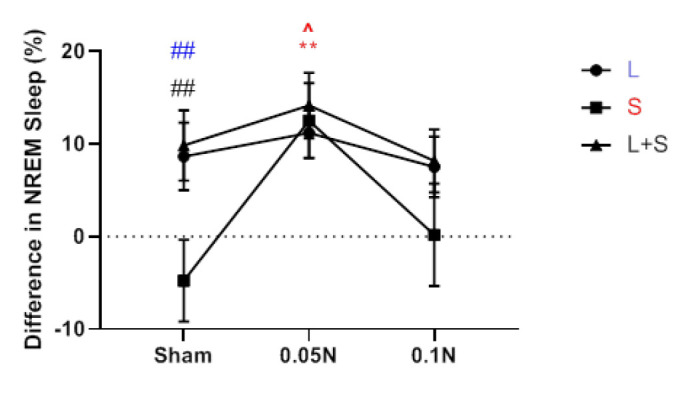
Comparison of the difference between NREM sleep values before and after the implementation of learning protocols (L) and stress (S) and learning with stress

**Figure 8:**
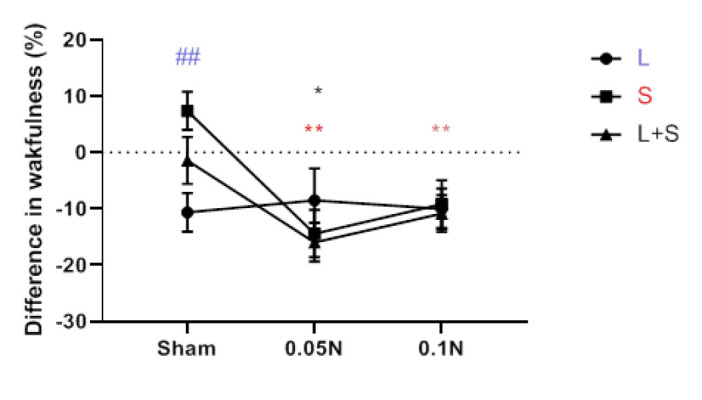
Comparison of the difference between waking values before and after the implementation of learning protocols (L) and stress (S) and learning with stress (L + S) in rat, as well as the difference in waking values with naloxone injection of 0.05 μg and 0.1 μg in these groups together (n=7)

According to a comparison of the values of the different groups, the increase in REM sleep brought on by learning was not more substantial than the rise in REM sleep brought on by the stress and learning protocol. The results revealed that, except for the group undergoing stress and learning protocols, REM sleep had no appreciable change between the two dosages of 0.05 g and 0.1 μg (Figure 6). The increase in NREM sleep at the two dosages of 0.05 mg and 0.1 mg naloxone amongst the various groups did not differ significantly from one another. However, examining the NREM sleep differences between multiple groups revealed that, except for the stress-only group, there was no discernible difference between the two naloxone dosages of 0.05 g and 0.1 μg. In the stress group (S), NREM sleep at a dose of 0.05 μg was significantly (*P*<0.01) higher than a dose of 0.1 μg naloxone (Figure 7). Comparison of the difference in wakefulness values between different groups showed no significant difference between the two doses of 0.05 μg and 0.1 μg naloxone in reducing the wakefulness rate between other groups. However, the decrease in wakefulness was significantly more notable in the learning group than in the stress group (Figure 8).

### Effects of naloxone on learning and memory during stress condition

**Figure 9:**
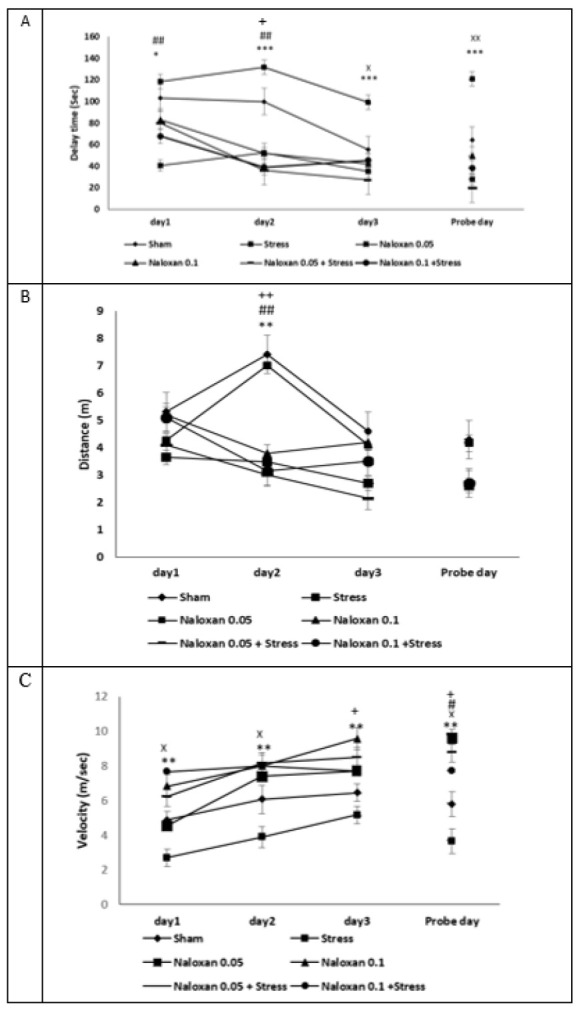
Effects of naloxone on the time delay in finding the target box in rat A, the distance traveled B, and the speed of finding the target box C in both doses of naloxone (n=7)

The results showed that stress increases the latency and reduces the movement speed to reach the goal on training days and the day of the memory test after 48 hr. The injection of both doses of naloxone minimizes the latency and increases the movement speed to reach the target. The memory test also observed this effect after 48 hr (Figure 9A, C). The 0.05 μg dose of naloxone reduced the distance traveled to reach the target on the second day (Figure 9B). 

### Effect of stress, learning, and naloxone on the expression of BDNF in the hippocampus

**Figure 10:**
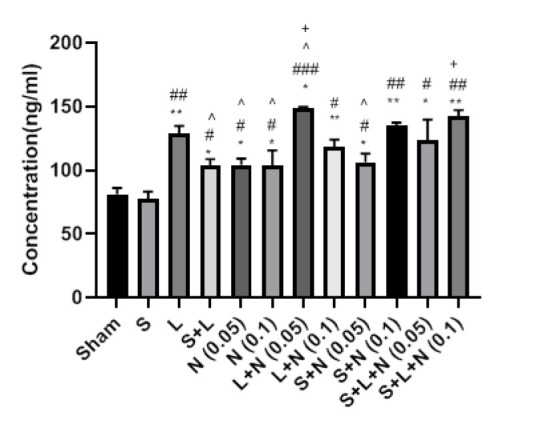
Effect of stress and naloxone on the expression of BDNF protein in the hippocampus of the rat. Both naloxone and naloxone learning boost BDNF levels. However, the increase in learning is greater than the effect of naloxone. Moreover, naloxone with a dose of 0.05 μg and learning have cumulative effects on BDNF levels. Even under stressful conditions, naloxone's effects on increasing BDNF are stable (n=7)

The results showed that stress reduces the expression of BDNF, though not significantly. In contrast, a significant increase in BDNF expression was observed in the hippocampus in rats on which the learning process was performed. Rats that experienced both learning and stress processes also showed increased BDNF expression compared to the Sham group. However, this increase was significantly lower than in the learning group. Also, injection of naloxone with both doses of 0.05 and 0.1 μg increased the expression of BDNF compared to the sham group. In rats that experienced the stress process with naloxone injection, the increase in BDNF expression was also observed when compared to the sham and stress groups. The highest increase in BDNF protein expression was observed in the group receiving naloxone 0.05 μg and included in the learning protocol. This increase in BDNF expression was significantly more outstanding in this group than in those who received naloxone alone or entered the learning protocol alone (Figure 10).

## Discussion

The results of this study indicated that multiple stress in the form of induction of immobility in adverse conditions, such as an uneven and rocky surface with tight pressure for two hours, reduces the amount of REM and NREM sleep and increases the waking time of the animal. In a 1995 study by Carrasco *et al*., the effect of immobility of one hour on sleep was reported with increased REM and NREM sleep (22). Moreover, another investigation by Rangi *et al*. examined the effects of physical and psychological stress (23). Interestingly, immobility increased REM sleep, especially in recording 6-hour sleep waves; after two hours of stress, REM sleep increased, while NREM sleep was unaffected (24). Furthermore, it has been found that immobility stress increased REM sleep in rats for two hours for three consecutive days but decreased REM sleep when they increased immobility time to four hours (25). According to these studies, stress affects rats’ sleep following its duration.

In a study by Erfani *et al.,* it was stated that immobility stress associated with a hard surface could change sleep stability by affecting the frequency and amplitude of the sleep spindle and also reduce the amount of REM sleep (26). In addition, it was shown that changes in sleep patterns in response to stress could be caused by variations in the hypothalamic-pituitary-adrenal axis (HPA axis) and neurotransmitters (27). Researchers reported that following sleep deprivation in rats, the corticotrophin-releasing hormone level increased along with the increased duration of wakefulness, while REM sleep decreased (28). Also, Palma *et al*. reported that one of the reasons for changing sleep patterns was a change in the activity of the HPA axis. However, REM sleep decreased up to 5 hr after physical stress, possibly due to the activation of neurotransmitters by physical stress. In addition to changes in the HPA axis activity, other significant factors such as serotonin, norepinephrine, and light create and regulate sleep (29, 30). 

The results of the current study on the effect of learning and memory using the Barnes maze on sleep show that the learning process during three consecutive days of training in the learning group naturally increases the duration of REM and NREM sleep when compared to the sham group. These increments in REM and NREM sleep could employ memory consolidation, as our results also indicated this memory improvement. One study suggested that the storage of recent information requires two steps. The first processing period occurs immediately after the acquisition, which is the encoding phase, and the second period occurs during REM sleep after learning, which is the consolidation phase. These two periods are characterized by increased brain activity. Another study stated that NREM sleep plays a role in initiation and REM sleep in memory transformation stabilization, and both periods of sleep play an important role in memory (31). Ackerman *et al*. indicated that NREM sleep plays a vital role in the memory stabilization process in the second phase. The researchers believed that NREM sleep enhances declarative memory and that REM sleep plays a role in stabilizing non-declarative and emotional memory (32). Another study found that an increase in sleep spindle activity was seen in the NREM phase following a rewarding learning test (33). The effects seen in the present study, which followed the instructions and approvals of the Medical Ethics Committee, were only due to our intervention (stress or learning). Therefore, increasing REM and NREM sleep levels confirm learning achievement and memory consolidation in the studied animals.

In addition, sleep before learning also plays a very important role in the learning and encoding process that takes place during waking. Research shows that stages 2 and 3 of sleep play an important role in enhancing our ability to learn, and some studies show that people who take a nap between learning periods increase their learning ability. It should also be noted that a lack of sleep makes it difficult to learn anything effectively, and sleep deprivation could induce learning disorders (34, 35). In other words, it can be said that sleep before learning plays an important role in encoding, and sleep after learning plays a role in memory consolidation. Furthermore, we can also mention the up and down theory in sleep, which refers to the removal of unwanted data and the weakening of unwanted synapses during sleep, thus creating an increase in the capacity of the brain to enter new data, especially in the hippocampus region (36).

This study implemented the Barnes maze to examine how stress affects memory and learning. The findings revealed that, in summary, rats in the sham group exhibited improved natural spatial cue learning after three days of training. Still, this ability declined when rats were exposed to stressful situations. The study also found that stress significantly reduced 48-hour memory compared to the control group. Studies have shown that stress has variable effects on different phases of the learning process. Hence, different types of stress in different situations have various effects on learning. For example, prolonged immobility stress can cause depression and decreased desire to move in the rat (37). A study stated that stress could affect all learning processes, such as coding, simulation, and memory retrieval. Pre-learning stress may alter coding processes or disrupt subsequent memorization (38). An article stated that two factors play a role in the process of memory during stress. The first is noradrenaline, which creates the emotional aspects of memory in the basolateral amygdala. The second factor is corticosteroids, which facilitate this process. However, if corticosteroids are released a few hours earlier, they can inhibit the amygdala and related behaviors. Therefore, a balance is established between noradrenaline and corticosterone to produce a response in the memory process (39). Increasing plasma cortisol levels following prolonged stress leads to decreased memory (40). In this research, the negative effect of stress on the learning process has been evaluated and reported. However, it seems that the animal has been able to compensate for the negative impact of stress, and this compensation can be due to the increase in NREM sleep in the learning process in the stress group.

The results of this study showed that injection of 0.05 μg naloxone in the basolateral amygdala increased REM sleep when compared to the sham group but did not significantly change NREM sleep and wakefulness. As well as REM, injection of 0.1 μg naloxone significantly increased NREM sleep. Also, in learning conditions, injection of naloxone (0.05 and 0.1 μg ) significantly increases REM and NREM sleep and decreases wakefulness duration in the animal compared to the learning group without naloxone injection. This part of the study showed that administering low doses of naloxone as an opioid receptor antagonist can improve sleep. This means inhibiting several opioid receptors in the BLA can reduce various environmental effects in inducing insomnia. This study demonstrates the importance of the opioid system in the BLA region to the physiological sleep of animals. It suggests that mu receptors may play a primary role, followed by kappa and delta receptors, which increase sleep duration by inhibiting them.

Interestingly, in stressful conditions, injection of naloxone (0.05 and 0.1 μg) in the BLA not only inhibited the effect of stress on REM and NREM sleep but also significantly increased REM and NREM sleep as well as reduced wakefulness duration. Stress appears to exert its effect on the induction of insomnia (at least in part) by stimulating the opioid system in the BLA region, which could be reduced by the injection of naloxone. The reduced effectiveness of high-dose naloxone compared to low-dose may also be attributed to its manifestation of opioid agonist effects at higher concentrations. In general, it can be said that due to the inhibitory role of opioid receptors and their impact on reducing sleep, the blockade of these receptors by naloxone could lead to increased REM and NREM sleep. Thus, the stress process may have reduced REM and NREM sleep by stimulating opioid receptors in the BLA region, which was prevented by naloxone injection.

It was shown in this study that the injection of naloxone in the BLA enhanced learning ability. In the stress group, naloxone not only eliminated the effect of increasing the time to reach the goal but also positively affected learning by reducing the time to reach the goal. It was also observed that injections of 0.05 μg and 0.1 μg in the stress group not only eliminated the effect of slowing down the speed of achieving the goal but also increased the speed of achieving the goal by possibly controlling depression. Liu *et al*. stated that stress reduces learning, and acute stress’s effect on different learning phases is different. Human and animal studies on the impact of internal opioids on the effect of stress on learning have shown that the release of internal opioids increases during stress. Hence, the internal opioid system mediates memory and learning (41). The results of an experiment performed on 15 C57 mice showed that forced swimming stress impairs information retrieval but has no adverse effect on memory acquisition and consolidation. In these animals, intraperitoneal administration of naloxone eliminated memory impairment. PCR results further showed that forced swimming reduced the level of mu-opioid receptor mRNA in the brain’s hippocampus, and administrating naloxone before stress reversed these changes. This study suggests that stress impairs cognitive memory recovery by opioid receptors (41). We can also consider the inhibitory effect of opioid receptors on REM sleep and the improvement of learning and memory by naloxone may be due to this effect (42) or morphine’s inhibitory effect on sleep-promoting neurons in the ventrolateral preoptic area (43).

The amount of BDNF in the hippocampus was decreased by the stress model utilized in this study; while not substantially different, the learning process considerably enhanced BDNF expression in the hippocampus. However, the amount of BDNF in the hippocampal region considerably rose in mice that were already under stressful circumstances before the beginning of the learning protocol (44, 45). In both stress- and non-stress-related situations, it was demonstrated that bilateral naloxone injections into the basolateral amygdala enhanced the quantity of BDNF in the hippocampus (46). Researchers have shown that the concentration of BDNF in the hippocampus decreases after stress, but this reduction returns to baseline after 24 hr. The BDNF level in the amygdala remains elevated for consecutive days, in line with the argument of the increased importance of the hippocampus in neurogenesis and neurodegeneration prevention (47, 48). This study observed no change in BDNF concentration in the stress group, possibly due to the interval between stress termination and hippocampal separation. It is believed that BDNF enhances CREB (Ca^2+^/calmodulin-dependent protein kinase) by affecting its receptors and stimulating the AKT, CAMK (cAMP-response element-binding protein), and protein kinase C pathways. It ultimately increases the expression of genes associated with cell survival, cell differentiation, and plasticity of syncytia. All of these activities indicate the critical role of BDNF in cellular stability, which is eliminated by stress (49).

Studies indicate the role of BDNF in sleep regulation in humans. People with no BDNF production have been found to have an NREM sleep disorder (50). Research has also shown that reduced sleep can lead to decreased BDNF production and secretion in the hippocampus. Some studies attributed this to reduced memory and learning ability and increased hippocampus degeneration due to insomnia (51, 52). The present work also found an interesting relationship between changes in sleep, memory, and learning with changes in BDNF levels. This positive correlation indicates that the BDNF of the hippocampus increased with the improvement of sleep induced by memory and learning protocols. Administration of naloxone by closing the window of opioid activity, primarily mu receptors in the BLA section, improved hippocampal function in memory and learning and increased the concentration of BDNF in the hippocampus (53). The hippocampus and the BLA have a very close anatomical and functional connection, and changes in basal neuronal activity in the hippocampus correspond to changes in basal neuronal activity in the BLA. It also even stimulates neurons in the hippocampus. Therefore, electrochemical changes in BLA following naloxone administration may also affect hippocampal activity. However, in this study, we did not investigate the changes in electrical activity in the hippocampus, which may have occurred with naloxone administration. This research suggests that the inactivity of the opioid system in BLA may play a positive role in mitigating the destructive effects of overactive BLA in the hippocampus. The role of gender was also a limitation of the current study. In view of the fact that stress-related and sleep disorders are more prevalent among women, it is proposed to investigate the interaction between stress, learning, and sleep in female rats.

S: Stress; L: Learning

Upper trace: EEG, middle: EMG, and lower trace: output results; green labels indicate NREM sleep, blue labels refer to REM sleep, and red labels refer to wake state.

MATLAB: Algorithm; EEG: Electroencephalogram; EMG: Electromyography

It also shows the injection of naloxone in two doses (0.05 μg and 0.1 μg) displayed next to each other. The two groups were compared using the paired t-test, which was found to be significant at the P<0.05 level. The stars indicate the significance of the results in both groups before and after the intervention (n=7).

* Shows a significant difference with baseline sleep (*P*<0.05), ** Shows a significant difference with baseline sleep (*P*<0.01), *** shows a significant difference with basal sleep (*P*<0.001)

REM: Rapid eye movement; NREM: Non-rapid eye movement

It also shows the injection of naloxone in two doses (0.05 μg and 0.1 μg) displayed next to each other. Both groups were compared using the paired t-test, which is significant at *P*<0.05. The stars indicate the significance of the results in both groups before and after the intervention (n=7).

* Shows a significant difference with baseline sleep (*P*<0.05), ** Shows a significant difference with baseline sleep (*P*<0.01), *** shows a significant difference with basal sleep (*P*<0.001).

REM: Rapid eye movement; NREM: Non-rapid eye movement

It was determined that there was a significant difference between the two groups using a paired t-test at the *P*<0.05. A star indicates significance in both groups before and after the intervention. It is also shown in this figure how naloxone drug injections at doses of 0.05 μg or 0.1 μg alone significantly increase NREM and REM sleep in two groups before and after injection. An increase in REM sleep at a dose of 0.05 is indicated (n = 7).

REM: Rapid eye movement; NREM: Non-rapid eye movement

# Shows a significant difference between learning + stress group (L + S) with stress (*P*<0.05)

** Indicates significant difference in learning + stress group (L + S) with naloxone 0.05 μg and without naloxone (*P*<0.01)

^ Shows a significant difference between the learning and stress groups in the two doses of naloxone (*P*<0.05)

*** shows a significant difference between the sham stress group and naloxone with stress (*P*<0.001)

## shows a significant difference between the learning group and the stress group (*P*<0.01)

REM: Rapid eye movement

(L + S), as well as the difference between NREM sleep values with naloxone injection of 0.05 μg and 0.1 μg in these groups with each other in rat (n=7)

## Indicates significant difference between learning + stress group (L + S) with sham stress (*P*<0.01)

^ Indicates a significant difference between the stress groups in the two doses of naloxone (*P*<0.05)

** Shows a significant difference between the sham stress group and naloxone with stress 0.05 μg (*P*<0.01)

## shows a significant difference between the learning group (L) and the stress group (S) (*P*<0.01)

NREM: Non-rapid eye movement

* Shows a significant difference between the learning + stress group (L + S) with naloxone and without naloxone 0.05 μg (*P*<0.05)

** Shows a significant difference between the stress group (S) and stress with naloxone (*P*<0.01)

## shows a significant difference between the learning group (L) and the stress group (S) (*P*<0.01)

× Indicates a significant difference between the stress group and the sham group

## shows a significant difference between the sham group and the naloxone group 0.05 μg

+ Shows a significant difference between the sham group and the naloxone group 0.1 μg

* Indicates a significant difference between the stress group and the naloxone and stress group

* Shows a significant difference with the sham group.

# Shows a significant difference with the stress group

^ Shows a significant difference with the learning group

+ Showed a significant difference with the naloxone group

BDNF: Brain-Derived Neurotrophic Factor

## Conclusion

In general, it seems that multiple immobility stress (psychological and physical) in our model leads to reduced REM and NREM sleep. On the other hand, since REM sleep creates the memory consolidation phase and NREM sleep strengthens the stabilization phase of memory formation, reducing these two stages and increasing the waking time due to stress reduces memory formation (54). According to our study, introducing stressed rats into the learning process reversed the negative effects of stress, specifically on sleep and memory, by increasing REM and NREM sleep and leading to memory strengthening. The present study showed that injection of naloxone increased REM and NREM sleep; it could also increase sleep duration effectively during stress and learning. Thus, naloxone could reduce the inhibitory effects of stress on memory formation.

Given that there are many opioid receptors in the BLA region which are related to the induction of stress. Inhibition of these opioid receptors in the BLA region or the corticosterone pathways by naloxone may directly reduce the effects of stress on REM and NREM sleep. On the other hand, due to bilateral neuronal connections between the BLA and hippocampal regions, BLA opioid receptors can affect the function of hippocampal neurons and lead to memory impairment. So, naloxone injection in this region by inhibiting opioid receptors and changes in intracellular pathways increases BDNF protein expression and improves the learning process and memory formation. 

In conclusion, considering the clinical implications, our study suggests that performing a learning exercise before sleep can be a good way to deal with daily stress and its negative effects on sleep. Secondly, inhibiting opioid receptors caused by naloxone administration reversed memory impairment and reduced stress-induced sleep periods. 

## Declaration

We have not used any AI tools or technologies to prepare this manuscript.
